# Complete Genome Sequences of Fribs8, Alyssamiracle, MakoManhole and DaviePasture, Four *Gordonia rubripertincta* Bacteriophages from Different Clusters Isolated from Soil in Davie, Florida

**DOI:** 10.17912/micropub.biology.001996

**Published:** 2026-06-26

**Authors:** Katie E. Crump, Janelle Bachman Rodriguez, Sarah Ballarin, Chloe Barreto-Massad, Taylor Butler, Sara Faruqui, Mansi Gandhi, Bianca Gonzalez, Megana Guntur, Christopher Huang, Anjaly Kappen, Brianna Khrakovsky, Elisabeth Loranger, Samantha Lopez, Kayle Mederos, Monserrat Montanez, Gabriella Nazarov, Ilan Nedjar, Oren Gaston Nedjar, Alyssa Ortiz, Naitik Patel, Shivani Patel, Vir Patel, Narindra Ramnath-Manohar, Anneliese Rodriguez, Joel Sanchez, Caleb Siguenza, Bhavya Soni, Autumn Taylor, Sadhika Uppalapati, Laurent Zayas Diaz, Kristin Parent, Sundharraman Subramanian, Julie Torruellas Garcia

**Affiliations:** 1 Biological Sciences, Nova Southeastern University, Fort Lauderdale, Florida, USA; 2 Biochemistry, Vanderbilt University, Nashville, Tennessee, USA; 3 Biochemistry and Molecular Biology, Michigan State University, East Lansing, Michigan, USA

## Abstract

We report on four Actinobacteriophages, Fribs8, Alyssamiracle, MakoManhole and DaviePasture, that were isolated from soil in Davie, Florida using *Gordonia rubripertincta* NRRL B-16540. Based on gene content similarity to phages in the Actinobacteriophage database, the phages were assigned to three distinct phage clusters: CT, DV, and DR.

**Figure 1. Virion morphologies of Gordonia rubripertincta NRRL bacteriophages (A) Alyssamiracle, (B) Fribs8, (C) DaviePasture and (D) MakoManhole:** All four phage exhibit a siphovirus morphology. Scale bar = 100 nm.

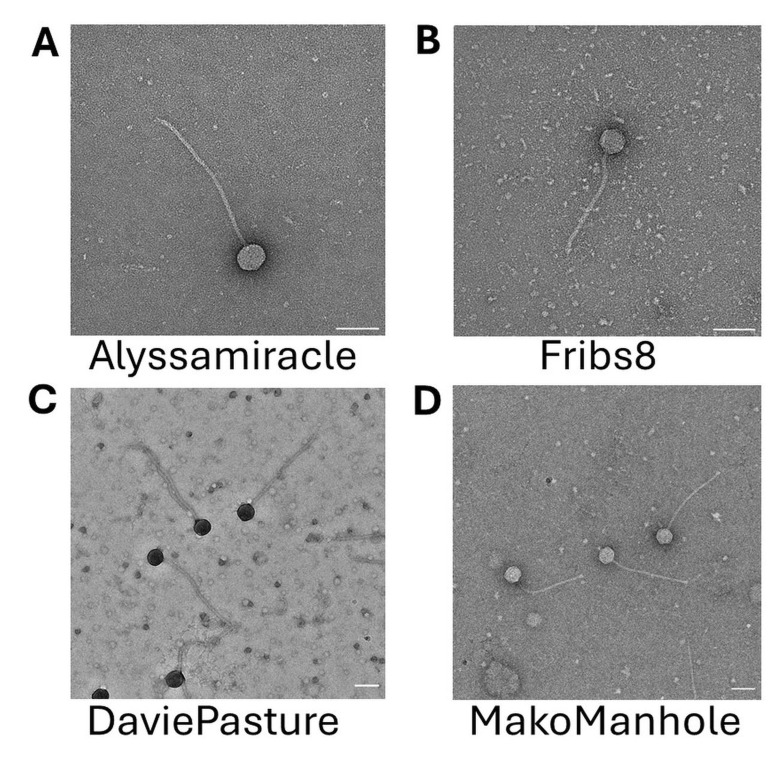



## Description

With an estimate of 10 ^31^ phage particles, bacteriophages are the most abundant biological entity on the planet (Comeau et al., 2008). While the phage population is vast, it is also diverse (Hatfull, 2015). Understanding the ubiquity and diversity of phages contributes to understanding how microbial communities are shaped (Koskella, Hernandez, & Wheatley, 2022). Here, we report the discovery of four new phages that infect *Gordonia rubripertincta* , a gram-positive soil bacterium. Fribs8, Alyssamiracle, MakoManhole, and DaviePasture were all isolated from soil in Davie, Florida.

All phage samples were isolated using standard methods (Zorawik, Jacobs-Sera, Freise, Sea, & Reddi, 2024). Samples were collected at Nova Southeastern University and in a cow pasture (Table 1; Davie, FL, USA). Each soil sample was washed separately in peptone-yeast-calcium (PYCa) medium. Then, the washes were filtered (0.22-µm pore size), and the filtrates were inoculated with *Gordonia rubripertincta* NRRL B-16540. Cultures were incubated with shaking at room temperature for 48h, and then filtered. The filtrates were each plated on soft top agar with *G. rubripertincta* overlayed on PYCa agar, yielding phages Fribs8, Alyssamiracle, MakoManhole, and DaviePasture. All bacteriophages were purified via three rounds of picking an isolated plaque and plating. All four phages exhibited small, clear plaques, which are predicted to be lytic phages after incubation at 30°C for 72 h. Five representative plaques were measured for each phage and their average diameters can be found in Table 1. High-titer phage lysates of each phage were prepared for transmission electron microscopy. Continuous carbon support film grids were glow discharged (PELCO easiGlow, 15 mA) for 45 s, then the samples were applied to the grids and incubated for 60 s. Next, the grids were washed with distilled water and stained with 1% aqueous uranyl acetate. The samples were imaged at the RTSF Cryo-EM Core Facility at Michigan State University using a Talos Arctica system operated at 200 keV. Micrographs were collected with a Ceta camera at a nominal magnification of 57,000 (1.78 Å/pixel) for A and B, and a nominal magnification of 45,000 (3.16Å/pixel) for C and D, with an exposure time of 1.0 s and a lens objective defocus setting of 5-mm under focus. Negative-staining transmission electron microscopy revealed siphovirus morphology for all (Figure 1). Capsid diameter and tail length for each phage are outlined in Table 1.

Table 1. Isolation Location, Plaque size, Phage Size, and Genome Data Information for Fribs8, Alyssamiracle, MakoManhole, and DaviePasture.  

**Table:** 

**Phage Name **	**Fribs8 **	**Alyssamiracle **	**MakoManhole **	**DaviePasture **
**Location **	26.07766N, 80.24469W	26.0807N, 80.24439W	26.07439N, 80.24096W	26.0436N, 80.24975W
**Plaque Diameter (mm)**	1.03 ±0.09 (n=5)	1.06 ±0.08 (n=5)	1.40 ±0.15 (n=5)	0.93 ±0.07 (n=5)
**Capsid Diameter (nm) **	59.2 ±0.74 (n=5)	64.3 ±1.31 (n=5)	70.34 ±0.82 (n=5)	70.53 ±0.90 (n=5)
**Tail Length (nm) **	266.7 ±10.3 (n=5)	364.5 ±14.4 (n=5)	322.89 ± 13.35 (n=5)	369.15 ±11.92 (n=5)
**Average Coverage **	328	864	644	1162
**No. of Reads **	223,945	393,501	264,461	523,794
**Length of Reads **	150-base single-end	150-base single-end	150-base single-end	150-base single-end
**Cluster **	CT	DV	DR	DV
**Genome Size **	45,985 bp	65,525 bp	61,592 bp	67,614 bp
**Genome Ends **	3’ Sticky Overhand	Circularly Permuted	Circularly Permuted	Circularly Permuted
**GC Content **	62.0%	57.6%	68.7%	58.4%
**No. of Genes **	67	97	83	98

Genomes of all four phages were auto-annotated using DNA Master v5.23.6 (Pope & Jacobs-Sera, 2018). GeneMark v2.5 (Besemer & Borodovsky, 2005), Glimmer v3.02 (Delcher, Bratke, Powers, & Salzberg, 2007) and Starterator v1.2 (Russell & Hatfull, 2017) were used to evaluate coding potential and start sites. Gene function was assessed using Phamerator using the Actino_draft database v578 (Cresawn et al., 2011), HHPred against the PDB_mmCIF70, Pfam-A, NCBI Conserved Domains databases, and UniProt-SwissProt databases (Gabler et al., 2020; Zimmermann et al., 2018) along with NCBI BLASTp using the Actinobacteriophage and NCBI non-redundant databases (Altschul, Gish, Miller, Myers, & Lipman, 1990). Transmembrane protein domains were determined using Deep TMHMM v1.0.24 (Krogh, Larsson, von Heijne, & Sonnhammer, 2001) and SOSUI (Hirokawa, Boon-Chieng, & Mitaku, 1998). tRNA genes were evaluated using tRNAscan-SE v2.0 (Lowe & Chan, 2016) and ARAGORN v1.2.38 (Laslett & Canback, 2004). No tRNA genes were identified in any of the four phages. Default settings were used for all software. 

The phages were assigned to three different phage clusters: CT, DV, and DR based on gene content similarity of at least 35% to phages in their respective clusters in the Actinobacteriophage database, PhagesDB (https://phagesdb.org) (Pope et al., 2017; Russell & Hatfull, 2017). Characteristic of other DV phages, Alyssamiracle and DaviePasture also exhibit rightward transcribed genes, with structure and assembly genes on the left arm and DNA metabolism and modification genes on the right arm of the genome (Torruellas Garcia et al., 2022). In contrast, DR (MakoManhole) and CT (Fribs8) phages show both rightward and leftward gene transcription. Like other DR phages, the first five genes in MakoManhole have 4-bp overlaps and encode for nucleotide modification enzymes which are thought to be associated with host evasion (Welsh et al., 2022). MakoManhole also conserves structural genes on the left arm of the genome and non-structural genes on the right arm (Versoza et al., 2022). Similarly, the first third of the genes associated with Fribs8 encodes for structure and assembly proteins, while the remaining genes are related to lysis and DNA metabolism, functions which are also observed in other CT phages (Simmons et al., 2025). No putative DNA modification enzymes were identified. Fribs8 was the only phage in this study to have a predicted -1 translational frameshift in the tail assembly chaperone. Interestingly, a phage genetically identical to Fribs8 was independently isolated by us from soil collected in Palm Beach Gardens, FL and named Evergreen22. Consistent with findings from other DV(Abdulrehman et al., 2025), DR, and CT (Simmons et al., 2025) phages, these phages do not contain integrase or immunity repressor genes suggesting they are incapable of establishing lysogeny. Taken together, this study highlights a continued compilation of diverse *Gordonia* phages (Pope et al., 2017).

This Whole Genome Shotgun project has been deposited in DDBJ/ENA/GenBank under the accession no. OR553910 Fribs8), OR475254 (Alyssamiracle), PQ184807 (MakoManhole), and PQ114740 (DaviePasture) (Table 1). Corresponding raw sequencing reads have been deposited in the NCBI Sequence Read Archive (SRA) under accession numbers SRX26306501 (Fribs8), SRX26306499 (Alyssamiracle), SRX26306502 (MakoManhole), and SRX26306500 (DaviePasture).
